# 3D VSP Imaging Using DAS Recording of P- and S-Waves in Vertical and Lateral Well Sections in West Texas

**DOI:** 10.3390/s24103044

**Published:** 2024-05-11

**Authors:** Yin-Kai Wang, Robert R. Stewart

**Affiliations:** Department of Earth and Atmospheric Sciences, University of Houston, 4300 Martin Luther King Blvd, Houston, TX 77204, USA; rrstewart@uh.edu

**Keywords:** distributed acoustic sensing, vertical seismic profile, seismic processing, seismic imaging, horizontal well

## Abstract

A 3D vertical seismic profiling (VSP) survey was acquired using a distributed acoustic sensing (DAS) system in the Permian Basin, West Texas. In total, 682 shot points from a pair of vibroseis units were recorded using optical fibers installed in a 9000 ft (2743 m) vertical part and 5000 ft (1524 m) horizontal reach of a well. Transmitted and reflected P, S, and converted waves were evident in the DAS data. From first-break P and S arrivals, we found average P-wave velocities of approximately 14,000 ft/s (4570 m/s) and S-wave velocities of 8800 ft/s (3000 m/s) in the deep section. We modified the conventional geophone VSP processing workflow and produced P–P reflection and P–S volumes derived from the well’s vertical section. The Wolfcamp formation can be seen in two 3D volumes (P–P and P–S) from the vertical section of the well. They cover an area of 3000 ft (914 m) in the north–south direction and 1500 ft (460 m) in the west–east direction. Time slices showed coherent reflections, especially at 1.7 s (~11,000 ft), which was interpreted as the bottom of the Wolfcamp formation. Vp/Vs values from 2300 ft (701 m) –8800 ft (2682 m) interval range were between 1.7 and 2.0. These first data provide baseline images to compare to follow-up surveys after hydraulic fracturing as well as potential usefulness in extracting elastic properties and providing further indications of fractured volumes.

## 1. Introduction

Optimizing fluid and gas flow in unconventional reservoirs as well as estimating ultimately recoverable resources are goals of seismic imaging and monitoring [1,2,3]. Thus, new and effective ways of recording and using seismic wavefields are of considerable interest. Fiber-optic sensing and utilization of the full elastic wavefield are promising technologies [4]. In 2017, the Apache Corporation undertook 3D/4D distributed acoustic sensing (DAS) VSP surveys to investigate whether time-lapse changes in travel time and amplitude can assist in understanding and improving hydraulic fracturing procedures. In particular, they wondered if there would be any differences detectable before and after 78 fracking stages [5]. Prior to the fracturing operation, a baseline 3D VSP consisting of 682 shot points was acquired using an optical fiber installed in a well and a DAS recording system. A grid of surface shots was designed and performed by two vibroseis sources. The main purpose of this full 3D survey was to image the stimulated reservoir volume (SRV), which is this project’s primary area of interest and provides the baseline image for follow-up hydraulic stimulations.

Regular DAS output measures the average variation of axial strain induced within a certain length (gauge length) of the fiber [6,7]. DAS is less sensitive to seismic wavefields as particle motion becomes more perpendicular to the fiber cable. The amplitude response versus incident angle of the DAS system was approximated as cos^2^(θ) for P-waves and sin(2θ) for S-waves [4,8,9]. Although DAS recordings of P-wave energy have been used to considerable advantage [10], there is also exciting potential for S-wave analysis, which promises valuable imaging results. We noted that S-waves have particle motions along the fiber’s sensitivity axis for certain arrival angles and, with shorter wavelengths, may provide higher resolution and attendant shorter gauge length processing. In addition, DAS generally records lower frequencies, allowing broader band assessments [11,12]. Since the incidence angle depends on the shot location and rock velocity, the directly arriving P-wave at the top few receivers is often largely perpendicular, so there is little direct P-wave arrival observed regardless of shot location. However, there can also be substantial refracted energy. S-waves and converted waves can be observed as the shot location moves farther away from the well. In addition to extracting both P- and S-wave events from the DAS, we are interested in the events captured by the DAS in the well’s horizontal portion. These details are described in the reflection sections.

In this paper, along with some standard processing of the vertical well and P-wave data, we aim to provide two additional contributions: an analysis of converted P-to-S events from DAS data and processing of DAS data in the horizontal part of the well. The ultimate goal was to use the complete set of elastic events captured by the well’s full geometry to provide augmented information for reservoir development.

## 2. Data Acquisition

**Figure 1:**
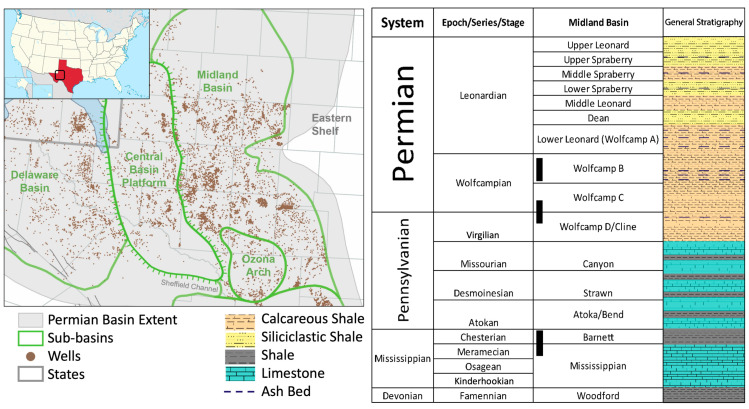
A structural map of the Permian Basin on the left (modified from [13]) and stratigraphic columns on the right (after [14]).

The DAS VSP survey was conducted in the Permian Basin, which includes several sub-regions and covers more than 75,000 square miles in West Texas and Southeast New Mexico (Figure 1). The Permian system comprises calcareous and siliciclastic shale. Although the precise survey location is confidential, the target formation is Wolfcamp’s organic-rich shale, which is divided into four benches: Wolfcamp A, B, C, and D. By combining stratigraphic columns and well logs collected around the DAS VSP well, a survey can be designed for target formations.

**Figure 2:**
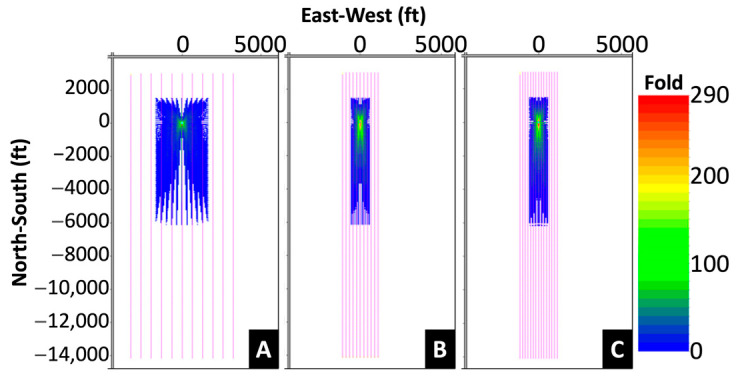
Comparison of three fold maps with a 55 × 55 ft (16.8 × 16.8 m) bin size. (**A**) Original, (**B**) narrow, and (**C**) 15 lines. The magenta lines represent the shooting lines (courtesy of Andrew Koller).

**Figure 3:**
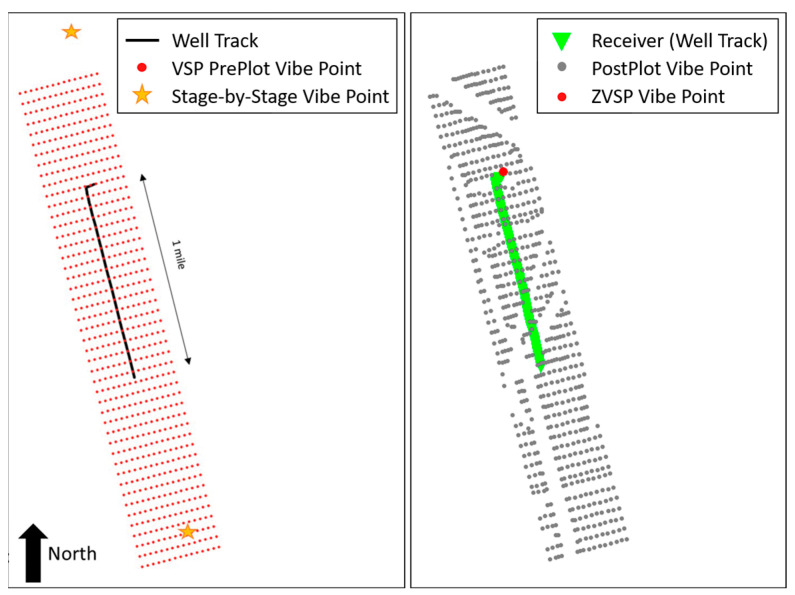
Plan view of the DAS VSP survey layout. The original survey design (modified from [5]) is on the (**left**). The final shooting grid (**right**), with gray dots representing the shot points and the green line representing the fiber location from above.

**Figure 4:**
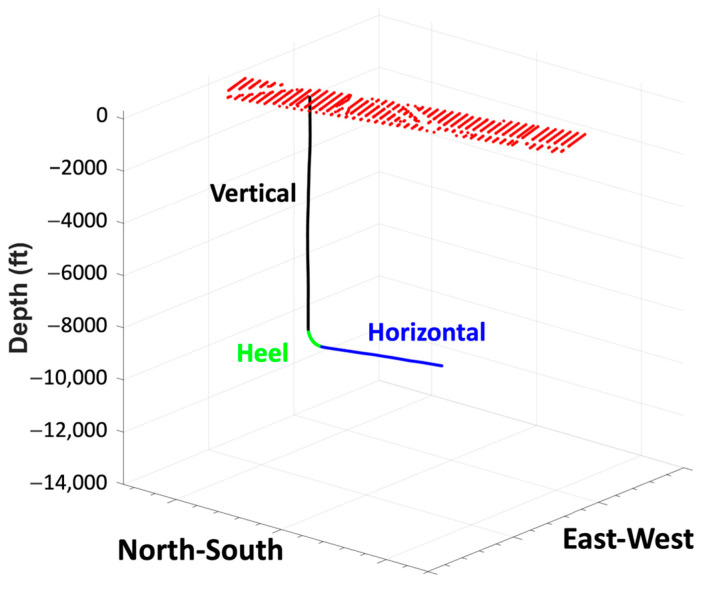
Diagram of the 3D VSP geometry. Shot points are marked by red dots. The well covers the vertical (black), heel (green), and horizontal sections (blue).

Allied Geophysical Laboratories (AGL) at the University of Houston and Apache Corp. developed the original survey design. Various shot spacings and areal coverages were tested synthetically. Figure 2 shows the fold maps of three proposed source distributions. In the first two proposed source grids, shooting lines and source locations were the same; however, the inline spacing in the second design was decreased. Both source grids consisted of 11 inlines and 79 crosslines with a total of 869 shots, and the second grid had a narrower source coverage. For the third proposed source grid, the number of inlines and crosslines was increased to 15 and decreased to 58, respectively. The spacing of both lines was adjusted to keep the total number of shot locations. Ultimately, a regular shot grid of 705 shot points [5] was recommended, which provided adequate coverage, trace density, and acceptable cost. The shooting grid comprised 15 inlines with 160 ft (~48.8 m) spacing and 47 crosslines with 300 ft (~91.4 m) spacing. Typically, some shot points in the field were moved or skipped with 682 shot points finally collected (Figure 3). At each shot location, two vertical vibes were used, and three 15 s linear sweeps from 4 to 80 Hz. The data were recorded for 4 s by the fiber optic cable along the well and processed with a gauge length of 16 m (~52.5 ft). Data were output at 13.3 ft (~4.1 m) intervals with a total of 1099 channels output. Figure 4 shows the geometry of the survey. The dataset was divided into three portions: vertical, heel, and horizontal sections, as recorded in the well. The data used in the first part of this paper were extracted from the vertical portion of the well and covered the first 664 channels down to a depth of 8817.9 ft (~2687.7 m).

## 3. Methods

**Figure 5:**
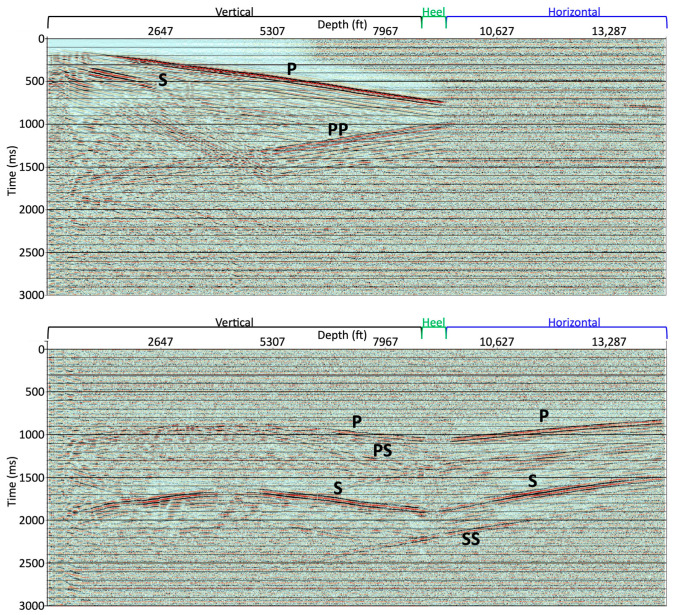
Two raw shot records for the whole vertical and horizontal well with two source offsets: (**top**) 1200 ft (366 m) and (**bottom**) 10,000 ft (3048 m) from the wellhead. Several interpreted events are annotated: direct P-wave (P), direct S-wave (S), downgoing P–S (PS), reflected P-wave (PP), and reflected S-wave (SS).

In this study, we used the vertical and horizontal sections of the well to record data. We also presented several examples of the shot records and our interpretation of some events. Each wavefield was observed in the shot records at different offsets given the varying sensitivity of the incident angle of DAS at the shot location (Figure 5). For the vertical portion of the well, DAS clearly recorded direct P-wave (annotated as P in the figure) and reflected P-wave (PP) from the near-offset shots. It recorded direct S-wave (S), downgoing P–S (PS), and reflected S-wave (SS) from the far-offset shots. For the horizontal portion of the well, the responses obtained from the near-offset shot were difficult to discriminate against the noise without further processing; however, the direct P- and S-waves, refracted P- and S-waves, and even converted S-waves were observed from the far-offset shots.

**Figure 6:**
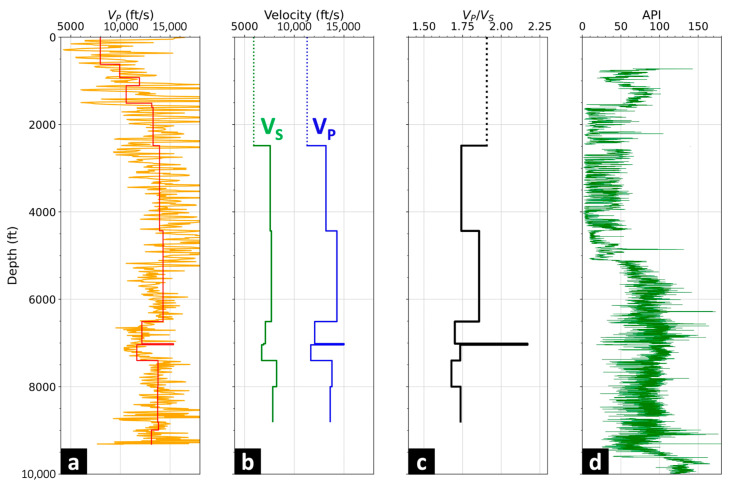
1D velocity and Q attenuation profiles. (**a**) P-wave velocity (red) calculated from the first-break DAS picks (orange); (**b**) velocity profiles of P-wave (blue) and S-wave (green) from a 2800 ft (853 m) offset shot record; (**c**) Vp/Vs curve calculated from the offset shot; (**d**) the gamma-ray curve.

We generated a 1D P-wave velocity profile from the first-break picks of the zero-offset shot. The Q attenuation was calculated by the spectral ratio method after applying the gauge length correction as outlined in [15,16]. Compared to the well logs derived from the well nearby, the velocity profile was edited to include major geological formations in the Permian Basin (Figure 6a). As the shot location moved away from the wellbore, downgoing S-waves could be observed in the records. Then, the Vp, Vs, and Vp/Vs profiles were generated from a 2800 ft (853 m) offset shot. Similar to creating the 1D VP profile, the velocity model was edited to highlight geological formations (Figure 6b). The apparent Vp/Vs was between 1.7 and 2.0 in this case and was used to generate a P–S common conversion point (CCP) profile (Figure 6c). Figure 6d shows the gamma-ray log, which indicates lithological changes and correlates velocity profiles.

**Figure 7:**
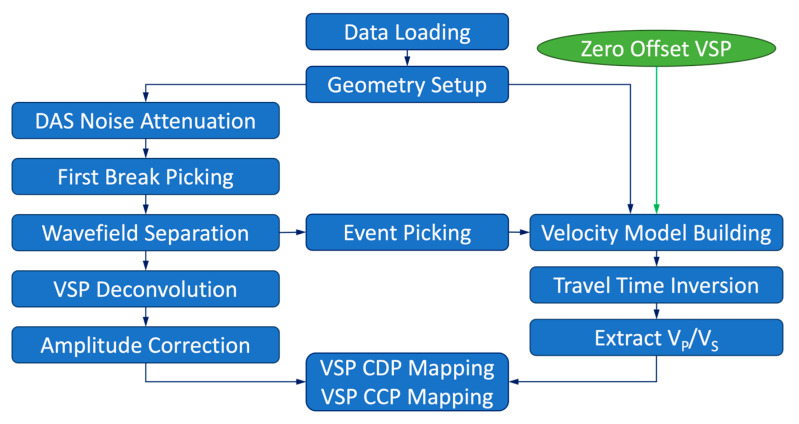
Processing workflow for DAS VSP. The amplitude correction was conducted only when spiking deconvolution or VSP deconvolution was applied.

The processing workflow (Figure 7) was slightly modified from conventional VSP procedures [17,18,19]. The main differences in the workflow between conventional geophone VSP and 3D DAS VSP were the use of common mode noise (CMN) removal (attenuating horizontal stripes likely caused by the interrogator unit; [20]), handling of substantially more traces, and not requiring 3C rotation operations.

The raw DAS records were severely contaminated by common mode noise (CMN), which appears as horizontal patterns in the records. To remove this type of noise while processing DAS VSP data, we applied a median filter along the depth to build the CMN model and subtracted it from the raw record. However, in the DAS records derived from the horizontal portion of the well, elastic events with high apparent velocity would have similar patterns in the shot record; therefore, we modeled common mode noise by stacking it across all channels for each shot record and subtracting it from the original input.

We analyzed a single shot line (inline 8), which is largely parallel but above the horizontal portion of the well to determine zero-, near-, mid-, and far-offset ranges based on dominating events in the shot records. Then, based on the results from the four single-shot VSPs, we applied parameters to all of the shots to create 3D P–P and P–S volumes.

At small-offset source locations, the high-amplitude tube wave can be observed in raw records. The apparent velocity of the tube wave (~4600 ft/s; ~1402 m/s) is relatively slower than the primary seismic event. Hence, we selected all records with offsets less than 1500 ft (457 m) and eliminated the tube wave by applying the median filter along its linear pattern across all channels.

## 4. Results

**Figure 8:**
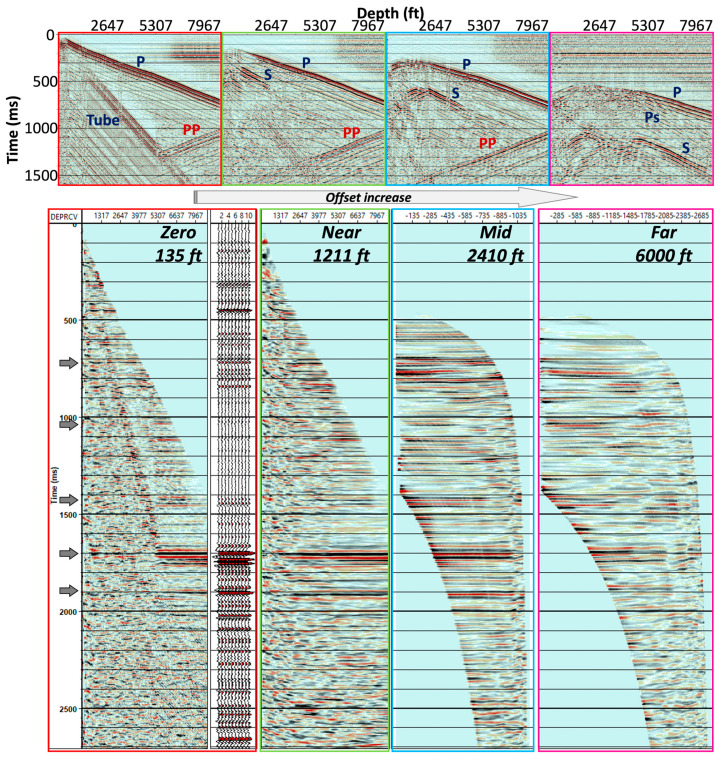
Four single-shot records (**top**) from offset sources and their resultant CDP profiles (**bottom**).

Four single-shot records were selected for initial analysis at 135 ft (41 m; zero-offset), 1211 ft (369 m; near-offset), 2410 ft (735 m; mid-offset), and 6000 ft (1829 m; far-offset) (Figure 8). The downgoing P-wave, upgoing P-wave (reflected), and tube wave were fairly straightforward to identify in the records. In this particular survey, the tube wave typically occurred within a 1000 ft (305 m) source offset and propagated at a velocity of approximately 4800 ft/s (1463 m/s). Short-offset records were severely contaminated by tube waves. Nonetheless, we reduced its effect via median filtering along the event as a preprocessing step. As the source offsets increased, the tube wave was reduced, and the refracted P-wave (upgoing) was recorded by channels close to the surface. Additionally, S-waves also became more apparent. We processed the vertical well data using the flow outlined in Figure 7. Four of the resultant common depth point (CDP) profiles are shown in Figure 8. Several reflections were observed, as indicated by gray arrows in the profiles. The reflections were masked at a shallow portion of the receiver depth when the shot points were near the wellhead.

The 3D VSP workflows with parameters designed from these four single shots were applied to the entire 3D DAS dataset. The 3D velocity model input was based on the 1D P-wave velocity profile from the zero-offset shot.

**Figure 9:**
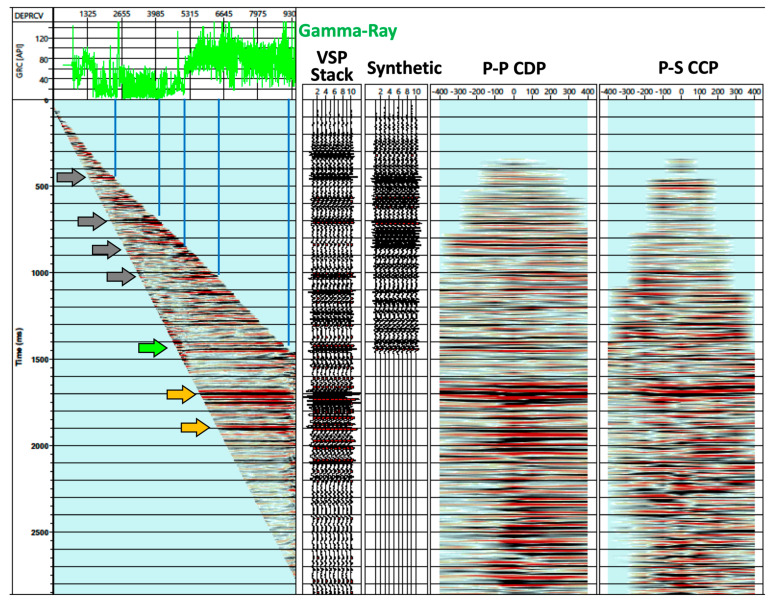
Composite plot including a gamma-ray log, near-offset VSP, zero-offset VSP extracted trace, synthetic seismogram, P–P CDP, and P–S CCP sections. The arrows and blue lines indicates correlated reflections with the well log.

Figure 9 shows a composite plot (L-plot) including the gamma-ray log, near-offset VSP, a stack of zero-offset VSP traces, synthetic seismogram, and P–P CDP and P–S CCP profiles. The VSP sections were extracted from an inline along the same orientation as the horizontal portion of the well. The correlation between gamma-rays and reflections indicates lithologic changes. Formations shallower than ~5100 ft (~1554 m) contain more sand than deeper formations. Notable reflections were observed in time slices between 1400 ms and 2000 ms in the region of the target lithologies. Based on the lithology logs acquired in the area, we interpreted the reflection at 1.4 s (green arrow) as the top of the Wolfcamp formation. Two reflections at 1.7 s and 1.9 s (yellow arrows) were interpreted as the bottom of the Wolfcamp formation and transitioning to formations beneath it, which predominantly consist of limestone.

**Figure 10:**
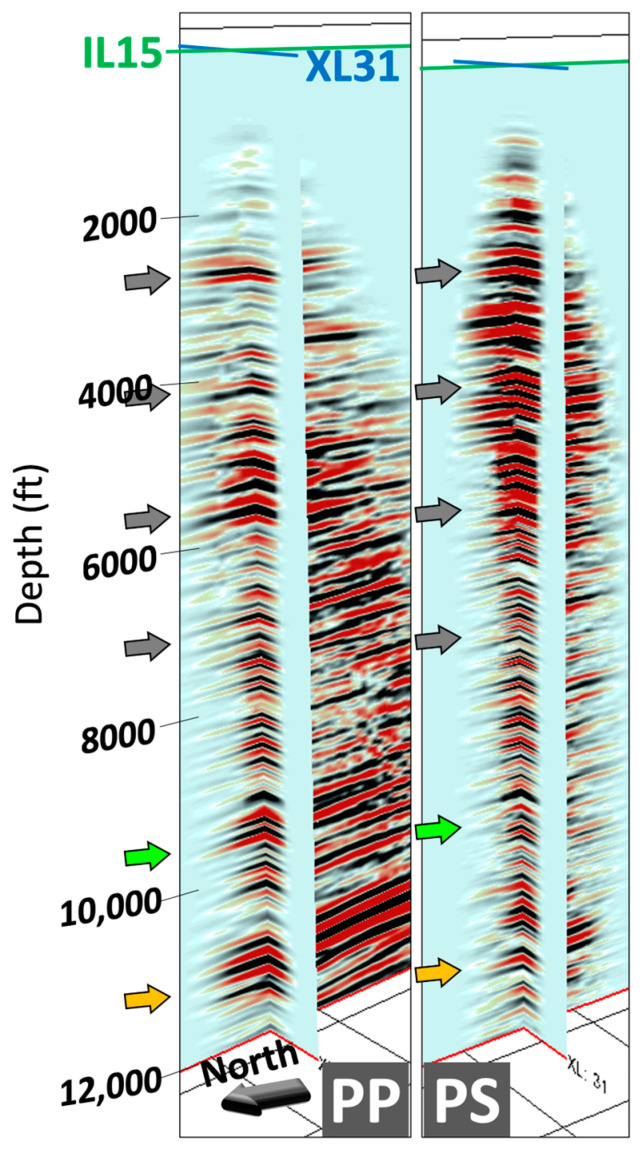
3D PP and PS volumes with inline (green line) and crossline (blue line) profiles. The intersection represents the well location.

**Figure 11:**
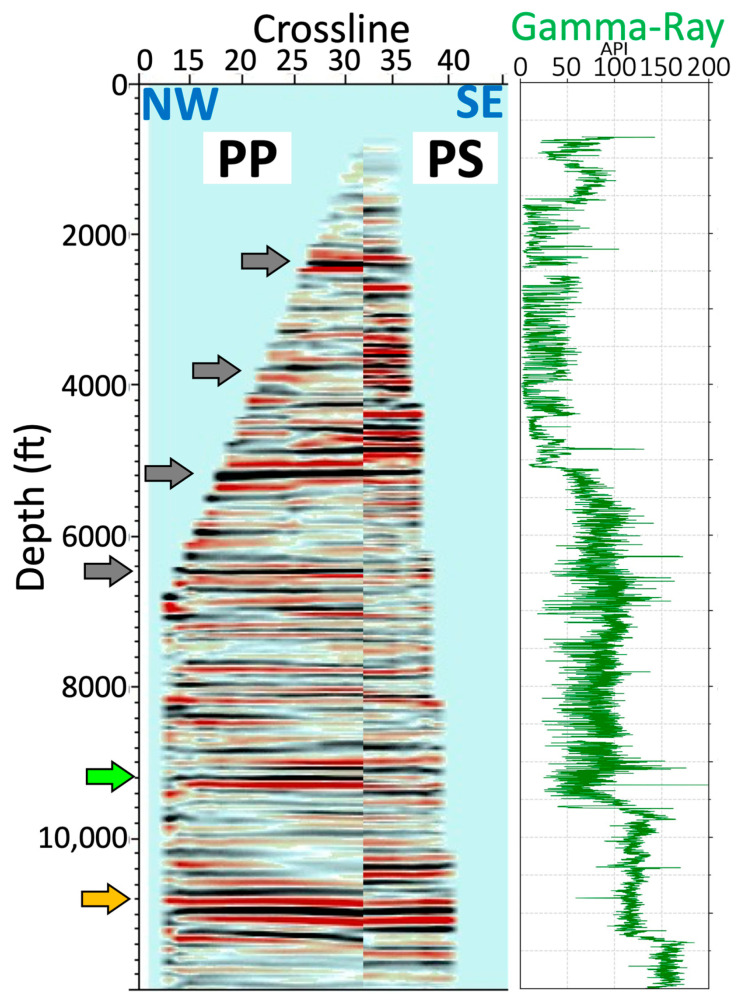
Comparison of PP, PS inline profiles, and the gamma-ray log.

Figure 10 shows two 3D P–P and P–S volumes after depth migration. The intersection of two inline (green line) and crossline (blue line) profiles marks the well location. The reflections extend to nearly 3500 ft (1067 m) in the inline direction and 1000 ft (305 m) in the crossline direction from the well. The entire 3D dataset provides substantial enhancement over the 2D sections. The comparison of two inline profiles extracted from P–P and P–S volumes is plotted in Figure 11. The first distinct reflection is at 2100 ft (640 m), which corresponds to the jump recorded around the same depth in the gamma-ray log. Groups of reflections (indicated by the arrows in the figures) with high amplitude are found in both PP and PS profiles down to 5500 ft. However, a few reflections from 6000 ft (1829 m) to 9000 ft (2743 m) in the P–S image are not as noticeable as in P–P. One notable reflection at around 11,000 ft (3353 m; yellow arrow) is correlated to both images, which we attribute to the bottom of the target formation.

**Figure 12:**
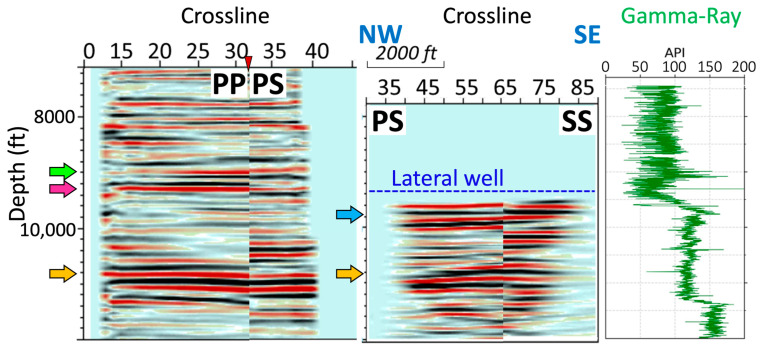
Comparison of inline profiles from the (**left**) VSP and (**middle**) horizontal well and (**right**) gamma-ray log. The blue dashed line represents the receiver array depth in the horizontal portion of the well. The arrows indicate notable reflections.

To obtain the image below the horizontal portion of the well, prior to the Kirchhoff depth migration, we first modeled the common mode noise by stacking it across the traces for each shot recorded and subtracting the noise model from the input. With the orientation of the DAS cable and the directionality of DAS sensors to the incident angle, the S-wave event, with the particle motion along the axial direction of the fiber cable, should be captured in the well’s horizontal portion. The geometry of the horizontal well and direct and reflected arrivals, both P and S, which have similar moveouts. The methods used to process VSP for wave separation cannot separate downgoing and upgoing events in this case. We proposed a scheme to eliminate much of the downgoing (e.g., the direct-P wave) energy from the raw record by muting the record at first arrival with a small time shift and a linear taper. The upgoing records were then applied to deconvolution with small window lengths. Figure 12 shows two inline profiles correlated to a gamma-ray log. The profiles extend the subsurface images another 2000 ft (610 m) in the inline direction. The blue dashed line represents the DAS receiver array depth in the horizontal section. The prominent reflection at around 9700 ft (2957 m; indicated by the blue arrow) was interpreted as the bottom of the stimulation reservoir volume area, which correlates to a significant jump in the gamma-ray log. Another major reflection was observed at around 11,000 ft (3353 m; indicated by the yellow arrow), interpreted as the bottom of the Wolfcamp formation. This finding bears some correlation to the P–P and P–S depth images from the vertical well section.

## 5. Discussion

In records with a source location close to the well, the tube wave dramatically and mysteriously diminished at a certain depth (~5307 ft), but the direct P-wave did not. We can imagine that this “cutoff” of the tube wave may be attributed to changes in the casing or tubing with which the fiber was cemented, to some kind of baffling, or some non-linearity in the interrogator system.

The original survey was designed to provide the desired lateral coverage. However, when the source offset was greater than about 5500 ft (1676 m) from the wellhead, the reflected P-wave was barely visible or no longer recorded due to the directionality of the DAS system on the horizontal reach. A similar issue occurred with the P–S event. The P–S CCP profile had narrower coverage than the P–P CDP due to a reflection point trajectory. Moreover, the incident angle of P–S was smaller than P–P, implying that its particle motion is nearly perpendicular to the fiber cable.

The main challenge in processing data from the lateral portion of the well is separating direct and reflected arrivals, both P and S, which have similar moveouts. The velocity model used in generating the depth image was first tested by applying conventional VSP Kirchhoff migration. The reflected events closest to the direct arrival migrate to the well position; hence, we muted the data at the first arrival plus time shifts to eliminate much of the downgoing energy. While the vertical section data did not appear to have obvious S–S reflections, the horizontal section did. Thus, we created an S–S section bearing some resemblance to the other images.

## 6. Conclusions

A 3D DAS VSP baseline survey was conducted in the Permian Basin of West Texas. A total of 682 shots covered the entire surface area of the horizontal well reach. In this paper, we processed and imaged a 3D VSP volume using P–P and converted wave (P-to-S) reflections. A composite plot shows the correlation between the gamma-ray and the PP and PS sections. Two 3D profiles obtained from the vertical section of the well imaging the target formation extended to 3500 ft (1067 m; inline) and 1000 ft (305 m; crossline) from the wellhead. Furthermore, P–S and S–S profiles from the horizontal portion of the well expanded another 2000 ft (610 m) from the VSP. The target formation at about 11,000 ft (1700 ms in P–P time) was imaged with P–P and P–S waves in VSP and P–S and S–S waves in the lateral well. The utility of DAS data is extended by establishing the presence of S-waves in fiber-optic data used for imaging. Furthermore, creating an image from the horizontal section of the DAS-equipped well provides an additional volume of coverage away from the wellhead. The correlated profiles provide further structure and lithologic information as well as the potential for more detailed follow-up elastic time-lapse imaging.

## Data Availability

The dataset presented in this article is confidential and not available.
